# The sands of time: Adolescents' temporal perceptions of peer relationships and autonomy in the context of living with chronic pain

**DOI:** 10.1002/pne2.12071

**Published:** 2022-01-26

**Authors:** Abigail Jones, Line Caes, Christopher Eccleston, Melanie Noel, Jeremy Gauntlett‐Gilbert, Abbie Jordan

**Affiliations:** ^1^ 1555 Department of Psychology University of Bath Bath UK; ^2^ 1555 Centre for Pain Research University of Bath Bath UK; ^3^ Division of Psychology Faculty of Natural Sciences University of Stirling Stirling UK; ^4^ 1555 Department of Health University of Bath Bath UK; ^5^ Department of Psychology Alberta Children's Hospital Research Institute Hotchkiss Brain Institute University of Calgary Calgary Alberta Canada; ^6^ Bath Centre for Pain Services Royal United Hospitals Bath UK; ^7^ Centre for Health and Clinical Research University of the West of England Bristol UK

**Keywords:** adolescence, chronic pain, development, longitudinal

## Abstract

The incidence of chronic and recurrent pain increases in adolescence. Prevalence of adolescent chronic pain is estimated to be 11%‐44%, with approximately 5% adolescents experiencing moderate‐to‐severe chronic pain. Adolescents with chronic pain also report unwanted changes in emotional, social, and developmental functioning. Very little is known about how adolescents with chronic pain make sense of their development, the role of pain in that development, and how such developmental trajectories progress over time. A multi‐methods qualitative study was designed to explore how adolescents make sense of their experience of chronic pain in the context of development. Nine adolescents (8 girls) aged 12‐22 years old (Mean = 15.7, SD = 2.8) were recruited from a UK national pain service. Adolescents completed an interview on entering the service, and a follow‐up interview 12 months later. They also completed monthly diaries in this 12‐month period. Data comprised 18 interviews and 60 diary entries, which were analyzed using inductive reflexive thematic analysis. Analyses generated one overarching theme entitled “tug of war: push and pull,” demonstrating developmental tension related to pain, and the cumulative impact these had over time. This overarching theme comprised two subthemes which capture these tensions across the developmental domains of peer relationships and autonomy. The first subtheme, “the shifting sands of peer relationships,” explores the ever‐changing closeness between self and peers. The second subtheme referred to “restricted choices” and how pain limited the participants' autonomy but that this, over time could push development forward. These results extend previous cross‐sectional research on the developmental consequences of chronic pain, showing the dynamic fluctuations and alterations to developmental trajectories over time.

## 1 INTRODUCTION

Adolescence, the period of transition between childhood and adulthood, is characterized by a number of key tasks such as the development of identity and autonomy, in addition to changes in the dynamics of peer relationships and their influence.^1^ Although everyday pain in childhood is common, it is often clinically uncomplicated.^2^, ^3^ Chronic pain, however, is pain that lasts for 3 months or longer and is associated with significant distress, disability, and developmental delay.^4^

The consequences of chronic pain on adolescents' social development have only recently garnered attention in the literature. Our definition of adolescence is an inclusive one, adopting the recent Lancet definition which extends into young adulthood (ages 10‐24).^5^ In part, this extension was to recognize that tasks of independence in the early to mid‐21st century have slowed compared to previous generations.^5^ This delay is particularly relevant to a chronic pain population for whom normative developmental milestones may take longer to achieve.^6^ However, evidence highlights how the picture might be more complex. Indeed, adolescents who have chronic pain report that they perceive themselves as being behind their peers in areas such as school progress and independence, but ahead of their peers in areas such as problem‐solving.^7^ In addition to self‐reported developmental differences as compared to their peers, adolescents who experience chronic pain also report alterations to their relationships with those peers.^8^

Although interesting, these observations are limited in their reach and ability to unravel the observed complexities. In a recent scoping review, we identified three key limitations of current evidence. First, there is a dominance of cross‐sectional approaches to studying social development, resulting in a lack of understanding as to how adolescent developmental functioning may change over time.^9^ Second, while there is a strong tradition of quantitative enquiry,^10^, ^11^, ^12^, ^13^ there is a lack of qualitative, idiographic analysis of the lived experiences, perceptions, and conceptualizations of adolescents who live with chronic pain. Although unusual, it is possible to undertake longitudinal qualitative research, achieved by conducting follow‐up interviews, which allow for reflection on changes over time,^14^ and diaries, which collect data on life as it unfolds.^15^ Such methods have been used individually with adolescents who experience chronic pain,^16^ and in an integrated manner with adults who live with chronic pain.^17^ Finally, our review identified a lack of research which explored the specific developmental domains (ie, peer relationships and autonomy) in detail, with many studies exploring social development or social functioning more broadly.

Consequently, to address the above‐identified gaps, the current research study aimed to answer the following research question, using a combination of interviews and diary methodology: how do the perceptions and experiences of adolescents who have chronic pain, in relation to their social development, change over the course of 12 months?

## 2 METHODS

### 2.1 Design

This study used a longitudinal design comprising semi‐structured interviews at two time points across a 12‐month period, and monthly online diaries over the same 12‐month period. This study presents findings from the data received from adolescent participants taking part in a wider project which also included interviews with parent participants. Parental data are not presented here to enable a sufficiently detailed analysis of the adolescent data.

### 2.2 Participants

Thirteen adolescents provided informed assent or consent and took part in the initial interviews; however, four were subsequently excluded for non‐completion of the follow‐up interviews and/or the diaries. Reasons for non‐completion were not given, participants either stated they did not wish to continue (n = 1) or did not complete the diaries and failed to respond to invitations to the follow‐up interview (n = 3). Consequently, the final sample of participants comprised nine adolescents (8 girls and 1 boy) aged from 12 to 22 years old at the point of consent (Mean = 15.7, SD = 2.8), all of whom described themselves as White British. Participants reported pain for a mean duration of 58 months (median = 37 months, range 15‐120 months, SD = 40.9), with a mean pain intensity of 6.25 (range 3‐9, SD = 2.1) at time of asking, on an 11‐point numerical pain rating scale. All participants were in full time education. See Table 1 for further participant details.

**TABLE 1:** Demographic details for adolescent participants (n = 9)

Pseudonym	Age (at consent)	Gender	Diaries (number completed)	Follow‐up interview	Self‐reported diagnosis	Outcome of assessment with national specialist pain service
Ellie	17	Girl	Yes (7)	Yes	Chronic coccyx pain	Received in‐patient pain rehabilitation treatment
Sarah	22	Girl	Yes (11)	Yes	Eczema and chronic neuropathic pain of the skin	Was offered, but declined in‐patient pain rehabilitation treatment
Stacey	16	Girl	No	Yes	Hypermobility	Received outpatient treatment from local secondary care providers
Laura	15	Girl	Yes (12)	Yes	Persistent localized leg pain	Received in‐patient pain rehabilitation treatment
Becky	13	Girl	No	Yes	Hypermobility and widespread chronic musculoskeletal pain	Received in‐patient pain rehabilitation treatment
Helen	12	Girl	No	Yes	Chronic ankle pain	Received outpatient treatment
Mark	16	Boy	Yes (6)	Yes	Hypermobility and chronic musculoskeletal pain	Not offered treatment, seeking private treatment
Suzi	15	Girl	Yes (12)	Yes	Chronic back pain	Received outpatient treatment
Joanna	15	Girl	Yes (12)	Yes	Chronic abdominal pain	Received outpatient treatment

### 2.3 Procedure

Following University of Bath (ref 18‐184) and UK National Health Service ethical approvals (IRAS 237547), potential participants were approached during their assessment for an NHS tertiary pain service in England (UK) between July 2018 and February 2019. Participants were approached if they were aged 11‐25 years old, assessed by the clinicians to have chronic pain (ie, pain lasting at least 3 months), and be sufficiently literate in use of the English language to be able to complete the study tasks. Interested participants were introduced to the researcher and completed assent or consent procedures via email and post. Eligibility criteria for inclusion in the final sample also included the completion of at least one of the two longitudinal elements of the study, that is, either completing the second interview or providing at least six diary entries (with at least one diary from each quarter of the year). Due to competing educational academic pressures, we did not require participants to complete all 12 diaries. The final data set comprised 60 diary entries and 18 interviews, providing a total of 78 sources of data. All participants completed both interviews, and six participants also completed at least six diaries, with at least one diary from each quarter of the year (mean = 10 diaries, range = 6‐12). Interviews were transcribed verbatim and checked for accuracy by an independent reviewer. During their time in the study, three participants received intensive pain rehabilitation, with the other participants either receiving outpatient treatment or no treatment from the NHS tertiary pain service. See Table 1 for further details regarding treatment. Most interview data, and all diary data, were collected prior to the COVID‐19 pandemic.

### 2.4 Measures

Participants completed basic demographic information and were interviewed remotely due to in‐person interviews being impractical given the geographically diverse participant sample. Telephone interviews were used as these provided the most accessible option for remote interviews at this point in time. After completing the interview, participants were sent an online qualitative diary for a period of 12 months. Online diaries took the form of a survey using Qualtrics (online survey tool, see *Qualtrics*)^18^. See Table 2, for examples of questions included in the interviews and diaries, with Appendices A, B, C‐C providing the full interview schedules and diary format. Following the final diary, participants were contacted to arrange the second interview where they re‐confirmed their assent/consent prior to the interview. Participants received a £15 online shopping voucher following the first interview, and a £20 voucher following completion of the diaries and second interview.

**TABLE 2:** Example questions from interview schedule and diary

Example interview questions (self and independence)	Example diary question (friendships and social life)
Can you tell me about what things make you, you? (Prompt) What interests do you have? (Prompt) What activities do you enjoy? (Prompt) How would you describe yourself? How does your pain affect how you feel about yourself? Can you tell me about the role of pain in terms of doing things that are important to you? Can you tell me about any ways your pain impacts your ability to make independent choices and decisions (eg, making a decision on your own about doing exercises, eating healthy, or taking medication)? Can you tell me about how much freedom you have to do the things you would like to do? (Prompt) Can you give me any examples of what this freedom is like? (Prompt) Can you tell me about times when you don't have as much freedom as you would like?	Friendships and Social Life In this section, we are asking about your experience of friends and social life over the past month. What contact have you had with your friends over the past month? (eg, what activities, spending time together, phone calls, social media, seeing at school/university/work) Can you tell us about times over the last month when your pain got in the way of friendships, and in what ways? How have you felt about your friendships over the last month?

The combination of interviews and diaries allowed us to gather rich, detailed data comprising both verbal and written responses, which detailed the participant's experiences throughout the course of a year. Diaries allow for the convenient near‐time personal record of difficult to access perceptions, including the opportunity for reflection before disclosure.^19^, ^20^ In contrast, the interviews gave participants the chance to reflect on their experiences overall, and on their past, present, and perceived future. The interviews also provided a more conversational style.^21^

### 2.5 Analytical approach

**FIGURE 1:**
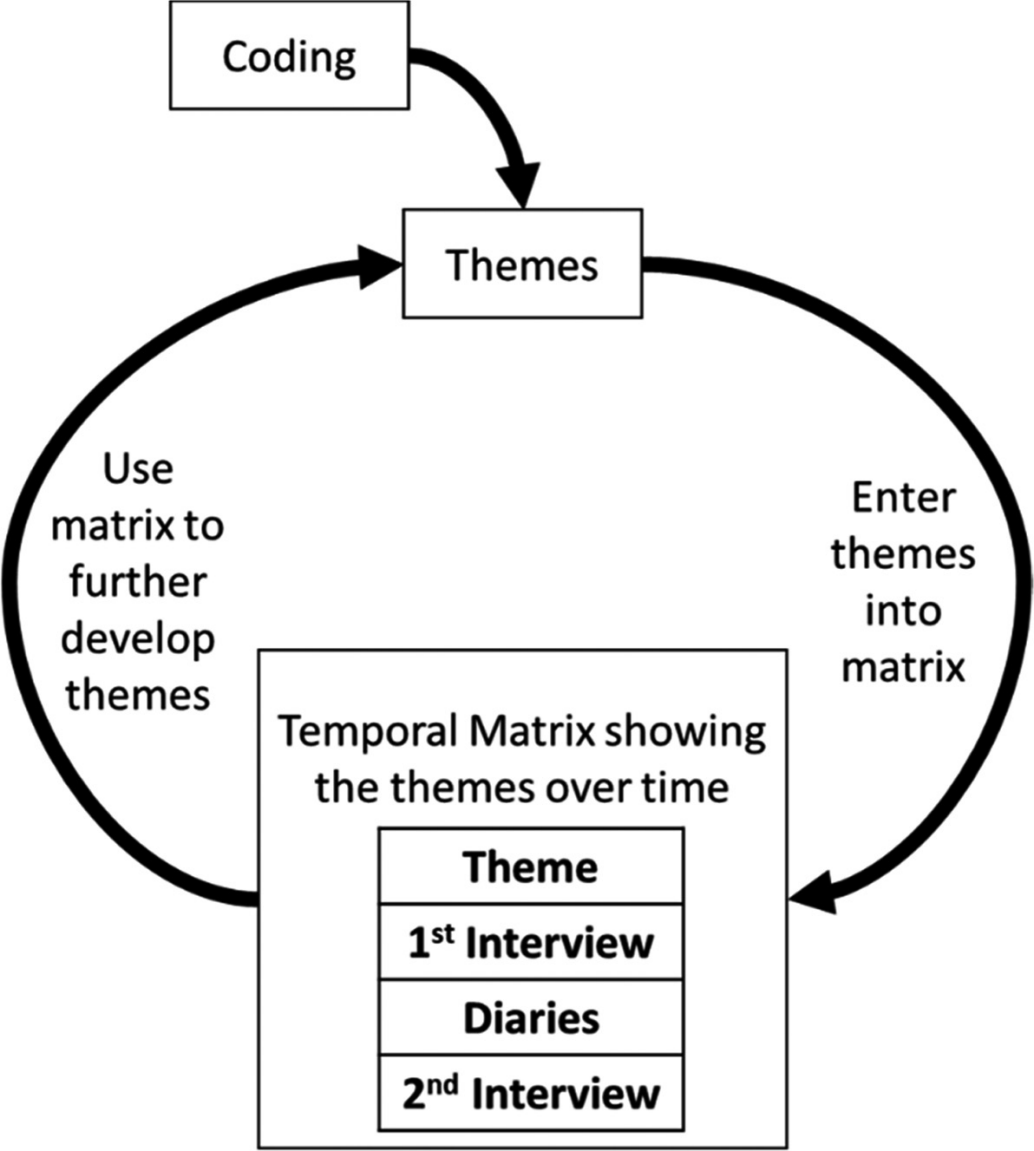
Trajectory analysis cycle used in theme development

We adopted a critical realist stance to analyses, which assumes an objective reality, accessible only through the subjective lens of experience and research.^22^ We used the inductive reflexive thematic analysis approach outlined by Braun and Clarke^23^, ^24^, ^25^ to analyze the interview and diary data. Reflexive thematic analysis has a well‐established history of use within a critical realist paradigm for the analysis of interview and diary data.^19^, ^22^ To address the temporal element of the research question around the possible change of social development over time, analyses incorporated Grossoehme and Lipstein's^26^ trajectory analysis as an additional final stage of the reflexive thematic analysis. This combination of analytic techniques was recently used by Simmons et al,^27^ in their longitudinal exploration of the experiences of youth mental health peer workers. The first author (AFJ) led this analysis by conducting the initial stages of data familiarization, inductive coding, and initial theme development. Initial coding and theme development took place using NVivo,^28^ and initial themes were then entered into the temporal matrix shown in Figure 1 (see Appendix D for worked examples). This temporal matrix demonstrated how the initial themes were represented throughout the data at each time point. In conjunction with discussions with two co‐authors (ALJ and LC), the temporal matrix was then used to further develop the themes, which were subsequently entered into a new matrix. We completed this iterative cycle several times, with each cycle refining the themes, which were then finalized through discussion with all authors.

As noted above, most data were collected prior to COVID‐19 and related restrictions. However, Helen's second interview was conducted very shortly after the implementation of UK lockdown restrictions which began in March 2020. In this interview, Helen was asked to focus on her experiences pre‐COVID, and distinctions were made between life “now” (ie, during lockdown) and “before” (ie, prior to lockdown). Despite the idiographic nature of this research, no special consideration of the impact of COVID‐19 on Helen's experience was made during the analysis due to the limited time she had spent in “lockdown” (less than one month), being insufficient in the context of the year‐long longitudinal nature of the research question.

### 2.6 Quality in qualitative research

Ensuring a high quality and robust analysis was a priority throughout, with several specific considerations relating to credibility, transferability, dependability, and confirmability. Credibility, which relates to the results being a fair representation of the phenomena under investigation,^29^ was partly achieved through the use of multiple sources of data. The use of two forms of data collection, and data collected at multiple time points increased our confidence in the accuracy of the record of participant experiences over time. Additionally, the data analysis process itself included credibility checks through the use of the trajectory matrices and co‐author checking at various points. Related to credibility, confirmability concerns the extent to which the results are grounded in the data,^29^ and therefore, quotations are included throughout. The quotations are taken from across the full range of participants, data types (ie, interviews and diaries), and time points. Details are provided to ensure the remaining two quality criteria of transferability, which relates to the extent to which results can be applied outside of the participant sample, and dependability, which relates to the research process being clear,^30^ are met. These details include appropriate contextualization of the sample and all quotations, and a clear description of the analytical process.

In addition to the considerations outlined above, reflexivity regarding the active role of the researcher is a key component of reflexive thematic analysis.^24^ To ensure that such reflexivity was present throughout the analysis, the first author (AFJ) had regular meetings with ALJ and LC to discuss the analysis. Themes were then finalized in discussion with all authors. As the first author (AFJ) recruited and interviewed all participants, reflexivity regarding this unique relationship with the data was considered throughout the analyses. Finally, reflexivity requires researchers to be aware of their position in relation to their participants and the data.^24^ Participants were approached at the point of assessment with a clinical team by the lead author, AFJ, a researcher with a background in psychology who is a mid‐30s white woman with no visible disabilities and who also conducted all data collection and was therefore the “face” of the study. In relation to author's positionality regarding the data, all authors are aware that due to circumstances (eg, age) we are not part a part of the group from which the data originates. Additionally, the research team has extensive experience in the scientific study of adolescent chronic pain.

## 3 RESULTS

Analyses of interview and diary data generated one overarching theme labeled: “Tug of war: push and pull,” which represented the adolescents' social perceptions of their development over the course of the 12 months. The theme shows that our participants experienced both positive and negative influences of chronic pain on their social developmental processes over time. Tensions between these positive and negative influences resulted in the adolescents feeling pushed and pulled in different directions, in a developmental tug of war that they could not seem to win. The theme comprises two subthemes: “the shifting sands of peer relationships” and “restricted choices.” Interestingly, despite both our research question and data collection focusing broadly on social development, participants elected to discuss issues and experiences specifically related to peer relationships and autonomy. The subthemes presented below are subsequently representative of the entire data set and reflect this participant‐selected focus on peer relationships and autonomy development when asked to describe their experiences of social development over time. The included quotations illustrate these subthemes, providing idiographic exemplars of how these subthemes were experienced by individual participants. For ease, quotations are italicized along with the participant's pseudonym, age at consent, and the nature of the data source (ie, first interview, a diary, or second interview).

### 3.1 “The shifting sands of peer relationships”

This subtheme explores changes which occurred in participant's relationships with their peers, and how they often lacked control over such changes. All participants reported loneliness as a result of their pain. For many, this was due to the time‐intensive and multi‐faceted nature of efforts adolescents made to self‐manage their pain, placing constraints on their physical and mental functioning. Sarah described in her monthly diary how managing pain requires substantial mental energy, resulting in a reduced ability to engage with her peers. Consequently, Sarah's experience of isolation was perceived as an unwanted yet necessary outcome of her efforts to manage her pain:

I don't particularly want to socialise when I'm in pain – I'd rather try and distract myself and try and forget about physical sensation by dully entertaining myself (for example, binge watching television/online series). It has made me feel very disconnected, but I didn't have the mental strength to do much about it. I know that once a bad period passes, they'll [friends] be supportive, knowing I didn't isolate myself on purpose, and I'll be able to socialise normally. But it is quite depressing in the midst of isolation, being alone quite literally with my pain. (Sarah, 22, month 3 diary)

As Sarah discusses, pain constricted opportunities to engage with friends and her wider peer group, leaving her feeling that pain is her only companion. Over time, this constriction can distort the dynamics of friendships and relationships with peers. However, meaningful engagement with friends requires more than the adolescents' mere physical presence. As Mark describes below, pain negatively influenced interactions with peers, even when he was present at school due to concerns about the burden that his pain could have on others:

My pain has stopped me from coming into school or being social in school. For example, I may be in a lot of pain and won't want to be a bother to anyone else. (Mark, 16, month two diary)

This isolation and constricted ability to engage with peers were common across many participants and did not occur in a single instance or event. Rather, the impact of these dual challenges of school absence and inability to be socially engaged when present had a cumulative impact which constricted friendships over time. Joanna, for example, struggled with this throughout her time in the study, describing in her initial interview that she had previously experienced difficulties with friends, as she felt excluded when she was absent from school:

It was…at the beginning of…year 10 [UK school age 14‐15 years]… that my friendship group changed, mainly because I wasn't in, either due to the IBS [irritable bowel syndrome], or because of the pain. Which meant I had to find other people that would still, almost include me and wouldn't exclude me if I wasn't there. (Joanna, 15, first interview)

Although Joanna was initially able to maintain these new friendships despite her absence, her diary entries reflect how they were not able to be maintained in the face of continued, recurrent absence:

I had quite a bit of time off and sometimes got isolated from the conversations when I was in… I feel that my friendships have been good, but could be better if I was in school more. (Joanna, 15, month four diary)

Joanna's experience highlights the needs for regular engagement with friends, and the importance of the school setting in facilitating such engagement. For some, continued absence from education placed strain on friendships, which in turn created alterations in the dynamics of friendships over time. Therefore, as Joanna discussed, some adolescents who experience chronic pain may be able to establish new friendships, but experience particular difficulty in maintaining these friendships. Extrapolating from these findings, it is possible that a reoccurring pattern of establishing, but not maintaining new friendships could develop, posing more long‐term challenges for relationship development.

In addition to experiencing challenges related to specific friendships, our participants reported concerns related to their relationships with their wider peer group. A key element of the divide between our participants and their peers was that their experience of pain and the effect it has may be difficult for others to understand, with the impact of this showing changes over time. In her first interview, Helen described how other children at school really “don't understand” her pain at all (Helen, 12, first interview). This lack of wider peer understanding of pain led to a sense of heightened self‐consciousness as Ellie described in her diary, which appeared to be particularly challenging for her at an earlier point in the study:

There have been points where it's been quite upsetting being around people that don't understand though. One of my flares this month I had to use a wheelchair and I felt extremely self‐conscious and un‐easy around people that didn't really know what was going on. (Ellie, 17, month two diary)

Adopting a temporal perspective, Ellie's understandings of these peer encounters developed as she began to understand that some challenging peer interactions were typical of normative everyday adolescent life rather than unique to her experience of living with chronic pain. Illustrating developments in maturity, over time Ellie altered her initial perceptions of these challenging adolescent encounters, reframing them to appreciate how poor peer behavior reflected badly on her peers rather than her own characters and behavior as she had initially perceived. Time was critical to these changes in perception and highlighted a sense of Ellie developing a greater sense of self‐assurance.

I did have a couple of groups [of school peers] that were quite difficult like I had um a wheelchair stored in one of the rooms in the sixth form block and there was one group that would always go in and take it and mess around on it outside and just quite disrespectful basically. But you're always going to get that anywhere you go, there's always going to be dickheads. So, you sort of got to deal with it. (Ellie, 17, second interview)

Ellie's experience demonstrates how the shifting sands of peer relationships contribute to the developmental “tug of war,” with the pulling back of development in one area, resulting in the pushing forward of development in another area. Many participants discussed how pain isolated them in some way, thereby pulling back their peer relationships. Interestingly, however, Suzi discussed in her second interview how being pulled back in other areas actually allowed her to spend more time with friends, thereby pushing her peer relationships forward and giving her what she perceived as a more normative adolescent experience:

I've stopped doing swimming and I've stopped doing running and that was partly because I was unwell and too ill to do it. But since then I haven't really wanted to go back to either of those sports… I'm with friends a lot more than I used to be. Umm, going out to parties and going out and things like that, and I'm able to do a lot more of those over the past year… I've been going out at night with friends and doing things I wouldn't have done before and probably still wouldn't do if I did loads of sport and things. I think it's enabled me to have more of a teenager experience in a way. (Suzi, 15. second interview)

### 3.2 “Restricted choices”

The second subtheme captures how participants reported that over time their pain reduced the choices available to them, leaving them feeling that the pain has control over their lives and futures. Participants discussed this lack of control in relation to both day‐to‐day activities (eg, going out with friends), and decisions which have a longer‐term impact (eg, school subject choices). Over the year, numerous separate such instances of reduced choice and/or control had a cumulative impact, resulting in the participant's autonomy development being pulled back. However, some participants learnt to overcome these restrictions, pushing forward their ability to make autonomous decisions. Such developmental progression was shown through advancement in their ability to be adaptable, take responsibility for themselves, and make difficult decisions. For example, in one of her first diary entries, Laura described how pain *“controlled most of my decisions over the past month.” (Laura, 15, month 2 diary)*. Interestingly, over time Laura's perceptions of the restricting nature of pain changed, with her later realizing (as reflected in her second interview) that despite the restrictions pain may impose, she could be an active agent in her own life.

I know my limitations so therefore I can be like ‘I can do this today’ or ‘I can't do this today’. So, say I wanted to work with my dad, if I really couldn't then I would say no, whereas last year, around the time we talked I wouldn't say no because I don't want to disappoint anyone or anything. But I feel like I can definitely make more of my own decisions. (Laura, 15, second interview)

As the above quotation illustrates, Laura's altered perceptions concerning the restrictive power of pain on her life highlight an increased sense of self and autonomy, and a growing assertiveness. As difficult circumstances with limited choices require more sophisticated autonomy skills in comparison with circumstances where there is greater choice, pain‐related restrictions may over time push development forward for some adolescents, leading to developmental maturity. This developmental progression of autonomy and personal agency, driven by pain‐imposed restrictions was also experienced by other participants, including Stacey, who discussed how her problem‐solving skills and adaptability developed over time. In her first interview, Stacey identified the need for adaptability, *“a lot of things I sometimes have to quickly adapt” (Stacey, 16, first interview)* yet did not elaborate on the specific nature of these adaptations or how she had achieved them. However, 12 months later, Stacey was able to describe the adaptations she makes in detail:

I've found… a lot of really cool gadgets that are helping me do things that I can do. I can open bottles and cans now because of a special thing that we've got. (Stacey, 16, second interview)

These quotations suggest that over time Stacey moved from identifying the need for adaptability, to developing specific techniques and methods to meet that need. Through this development of adaptability and problem‐solving skills, Stacey used the pulling back of her choice and control imposed on her by her pain, to push forward her autonomy.

Beyond the pain itself and the restrictions it imposes, many participants described wider social pulls on their autonomy. Parental wishes and behavior and the conflicts this created between adolescent and parent regarding protection from further pain and associated disability were particularly highlighted. In her first interview, Becky discussed this incongruence as reflected in her and her mother's differing perspectives on activities which may trigger pain:

Sometimes when I want to do something and Mum will go no, no, you can't cos of your condition… Sometimes we'll be out, and I will want to go somewhere like on a ride, but Mum will say no because last time you injured yourself… It's not going to change anything if you stay at home doing nothing. It's just going to make you a bit depressed. I'm always in pain, so I might as well just get out (Becky, 13, first interview)

Becky reflected on this further in her second interview, explaining that despite improvements in her pain, her mum still worried. However, Becky also expressed the insight that although the specific nature of her Mum's concerns may have changed, the level of her mum's concern was not more than what her peers' parents may have had:

To be honest, if Mum wasn't nagging it wouldn't be Mum… I think it's quite normal, it's just what mum nags me about is a bit different than normal. (Becky, 13, second interview)

Becky's reflections here suggest that there may be multiple factors which contribute to autonomy being pulled back. First, there is the pain itself which limits their choice and control. Second, parental concerns and protective behaviors may place additional pulls on the choices available to the adolescents, with such a focus on pain potentially increasing the sense of difference between the adolescent and their peers. Finally, adolescents may not be able to accurately assess the autonomy enjoyed by their peers who do not experience chronic pain, and this, combined with their perceptions of their parents' protective behaviors, may lead them to perceive greater pulls to their autonomy. Overcoming such perceptions may help adolescents to develop their own agency and more sophisticated autonomous skills.

## 4 DISCUSSION

This novel study, which comprises both interview and longitudinal diary data from adolescents who live with chronic pain, has shown that over time, such adolescents may face both a restriction in the choices available to them, and a gap between them and their friends and peers. This “gap” can fluctuate in size, widening and narrowing over time, and may be related to the restriction of choices for personal development. These results reflect previous findings related to associations between chronic pain conditions and problems related to adolescent social development.^8^, ^31^ The social gap and associated difficulties experienced by adolescents living with chronic pain exist with both their specific friendships and relationships with their wider peer group. Such findings are congruent with previous work which suggest that adolescents who live with chronic pain participate in fewer peer activities, have fewer friends, are more isolated, and have different perceptions of their friendships as compared with their healthy peers (Forgeron et al).^32^, ^33^ These difficulties are important to note as research has shown that relationships with friends and peers become increasingly important during the normative transitional processes of adolescence with peer approval and acceptance becoming especially valued by adolescents.^34^, ^35^ The findings presented here add a much‐needed longitudinal perspective to this area of study, suggesting that the perceived “gap” between adolescents who experience chronic pain, and their friends and peers fluctuate over time rather than existing as a static entity. The fact that this “gap” changes temporarily is positive, suggesting there are ways in which adolescents can be supported to bridge the gap and create strong social connections to friends and peers. However, the “shifting sands” of friendships and peer relationships may make them harder to navigate, as such changes may necessitate a constant change of approach, which adolescents may or may not be able to accommodate.

Our findings also reflect the results of previous research, which suggest that chronic pain is associated with restrictions to adolescents' choices and autonomy development^9^ while adding a longitudinal perspective. Although participants generally viewed pain as presenting developmental challenges, they also identified areas of developmental enhancement as a result of pain necessitating their engagement in complex developmental processes. Such gains may be due to some developmental areas requiring an individual to experience some form of hardship which facilitates learning and growth. This finding is congruent with a growing body of research showing developmental advantages associated with chronic pain.^6^, ^7^, ^13^ Interestingly, tension between pushes and pulls may exist both within individual developmental domains, and across different domains. An example of this tension within a developmental domain is the restriction of choice as this may initially be felt as a pull on autonomy but may in turn push forward autonomy development for some adolescents. An example of the tension between pushes and pulls across different developmental domains is how such a pushing forward of autonomy may in turn widen the gap with peers therefore leading to further constriction of peer relationships. Therefore, although individual developmental domains are in many ways distinct from each other, they are not independent from each other and so the exploration of one domain must incorporate consideration of the related domains.

Additional important insights provided by our study findings include the importance of addressing the broader social context when assessing and treating adolescents who live with chronic pain. Extending the results of existing literature,^9^, ^36^ our study findings reinforce the importance of considering the complex interactions which may occur between different elements of the social context, such as peer relationships and educational attendance. Specifically, this study adds a temporal exploration to the literature, revealing an ever‐changing picture of peer relationships. This longitudinal work builds on previous work which has explored the ways in which children and parents reminisce about past pain,^37^, ^38^ and work exploring the future narratives of adolescents who have Complex Regional Pain Syndrome.^39^ Together such research is building an evidence base which explores the narrative timelines of pain. Currently, questions remain regarding how best to support the social developmental trajectories of adolescents who experience chronic pain, and whether there are specific ages or developmental stages which are particularly sensitive to pain‐related disruption or intervention.

Clinically, when considering most appropriate treatment options, it may be helpful for clinicians to consider the pressures that the “tug of war” identified in this study puts on adolescents' engagement with social development when living with chronic pain. Additionally, it is interesting to note that some participants reported using strategies contrary to those recommended by pain treatment programs (eg, reducing activity, self‐isolation) to manage their social development in the context of their pain. Therefore, clinicians may find it helpful to adopt a developmentally informed approach as this may provide important insight into sometimes counter‐intuitive priorities and motivations of adolescents. Developmental maturity or other developmental factors may also impact the degree to which adolescents are able to use the skills and techniques they learn in treatment. As developmental trajectories may not be linear, adolescent's benefits from treatment may similarly fluctuate. Our results also suggest that treatment options which also provide social opportunities, such as peer support groups, may help adolescents overcome both clinical and developmental challenges. This conclusion fits well with previous research which has explored a peer mentorship program for adolescents who have Juvenile Idiopathic Arthritis (Ahola Kohut et al; Stinson et al).^40^, ^41^ This link between treatment and social development is an important focus for future research with such insights having both clinical and developmental implications. Another avenue for future research is exploring the multiple factors, such as parental focus on pain, which may restrict adolescent autonomy development. Such research could explore what interventions may support adolescents to navigate their autonomy development in the context of chronic pain, and how parents and other significant others can provide developmentally appropriate support. Finally, future research is needed to explore in more detail the relationship between the timing of interventions and an adolescent's age, developmental stage, and duration of pain.

The learnings from this study are limited by its methods and approach. First, as in most chronic pain research,^9^, ^11^ the participants were primarily girls and were all from a homogenous ethnic and linguistic (English as a first language) group. In addition, the focus on the perspectives of adolescents themselves resulted in a lack of sampling of other important perspectives such as those of parents, teachers, and clinicians. Second, although the extended definition of adolescence proposed by Sawyer et al^5^ is appropriate, this resulted in a sample with participants aged 12‐22 years old. Therefore, there is substantial variation in the developmental stage, concerns, and experiences of our participants, resulting in a lack of clarity around the specific concerns at each developmental stage. However, given the longitudinal and developmental focus of this study, the wide age range of the participants allows us to explore concerns which are saliant across developmental stages. Third, this study is limited due to participant attrition and three participants not completing any diaries. Given the length of follow‐up and demands of data collection, it is unsurprising that there would be participant attrition and most participants would not be able to complete all the diaries. However, the self‐selection of participants means that there may be elements of the social development of those who withdrew which remains uncaptured by this study. Due to limited comparable literature in the adolescent chronic pain field, it is difficult to assess if this level of attrition and missing data are in line with other studies. We recommend that future studies consider the role of rapport building in the retention of participants, and how data collection may be impacted by school holidays and examination periods. Finally, despite the participants having a range of treatment experiences throughout their time in the study, we did not explore how such experiences may be related to social developmental trajectories.

Through the novel use of a combination of interviews and diaries over the course of 12 months, this study provides an important longitudinal, idiographic exploration of adolescent social development in the context of chronic pain. Future research is needed to extend this understanding, specifically the complexity of developmental gains and ways in which adolescents might develop despite chronic pain, and how these personal gains might also present further struggles for their social development. Further, the clinical implications of this non‐linear development trajectory should be considered.

## CONFLICT OF INTEREST

The authors report no conflicts of interest.

## APPENDIX A

### BASELINE INTERVIEW SCHEDULE

**Table:**

Introduction	Hello, I am Abbie Jones. I am PhD student at the University of Bath looking at the impact of chronic pain on young people. As part of this study, I am interviewing young people who experience pain, and their families. We will complete an interview with you now, and again in a years' time. You have given your consent to take part in this study, but you are welcome to withdraw at any time and without giving a reason. Taking part does not have an effect on the clinical care you receive, and I will not share any information you give me with the clinical team. However, if you tell me something which makes me concerned for the safety of yourself or someone else, I will need to pass this on to appropriate people, and I will tell you if I need to do that. This interview will last approximately 30‐50 min, but we can stop at any time, or have breaks if you would like. If you don't understand a question, please tell me. If, I ask you a question that you do not want to answer, please do let me know and I will move onto the next one. Is there anything you would like to ask at this point?
Getting started	Can you tell me about how your pain affects you? What's your diagnosis? When were you diagnosed? Can you give me a brief time line of your pain?
Self and independence	Can you tell me about what things make you, you? What interests do you have? What activities do you enjoy? How would you describe yourself? How does your pain affect how you feel about yourself? Can you tell me about the role of pain in terms of you from doing things that are important to you? Can you tell me about any ways your pain impacts your ability to make independent choices and decisions (eg, making a decision on your own about doing exercises or eating healthy or taking medication)? Can you tell me about how much freedom you have to do the things you would like to do? Can you give me any examples of what this freedom is like? Can you tell me about times when you don't have as much freedom as you would like?
Future	What do you think your future looks like? What things would you like to do over the next 5 years? Can you tell me about any ways you feel your pain may affect these plans?
Family/Home	Can you describe your family life to me? Who lives at home with you? How often do you see your parents/siblings (if not living with them) What does your relationship with your parents look like? What does your relationship with your siblings look like? What kind of activities do you do together with your parents/siblings? What do you tend to talk about with your parents/siblings? Do you feel like these relationships/activities/conversations have changed since the start of your pain? What things do you do at home? Can you tell me about any chores you do? Thinking about other people your age, in what ways do you think your home life is similar/different? How do your family/housemates/partner react to your pain? Do you find this helpful/unhelpful, why/how? Can you tell me about how these things we have discussed about you at home have changed over time? For example, how have things changed since this time last year?
Friends and relationships	Now I would like to talk about friendships and other relationships. Can you tell me about any friends that you have? Can you tell me for you, what makes a good friend? How do you feel about your friendships? What do you like doing with your friends? Can you tell me about any ways in which pain may influence your friendships? How do your friends react to your pain? How did you become friends with them? How do you spend time with your friends? What do you like doing with your friends? How do you communicate with your friends? (eg, social media) What does your relationship with friends look like when you are experiencing pain? How do your friends react when this happens? How do you feel about your romantic relationships? Can you tell me about any ways in which your pain affects your relationships? How does your partner react to your pain? Can you tell me about any ways in which you feel your relationship is different from couples where chronic pain is not an issue?
School/Work	Can you tell me about school/education/employment? What kind of school/education/employment are you in? How do you feel you are doing at school/education/work? How do your teachers/employer react to your pain? How do your peers/colleagues react to your pain?
Close	Is there anything else you would like to tell me about your pain and your life? Thank you for taking part in this study, your answers are very useful in helping us to better understand how pain impacts the lives of young people. I will be in touch over email about the monthly diaries. Do you have any questions about the interview, the diaries, or anything else?

## APPENDIX B

### FOLLOW‐UP INTERVIEW SCHEDULE

**Table:**

Introduction	Hello, as you will remember from last year, I am Abbie Jones, a PhD student at the University of Bath looking at the impact of chronic pain on young people. As part of this study, I am interviewing young people who experience pain, and their families. We completed an interview with you a year ago, and this is the follow‐up interview. You have given your consent to take part in this study, but you are welcome to withdraw at any time and without giving a reason. Taking part does not have an effect on the clinical care you receive, and I will not share any information you give me with the clinical team. However, if you tell me something which makes me concerned for the safety of yourself or someone else, I will need to pass this on to appropriate people, and I will tell you if I need to do that. This interview will last approximately 30‐50 min, but we can stop at any time, or have breaks if you would like. If you don't understand a question, please tell me. If, I ask you a question that you do not want to answer, please do let me know and I will move onto the next one. Is there anything you would like to ask at this point?
Getting started	Can you tell me about how your pain has affected you over the last year? What's your diagnosis, has this changed? When were you diagnosed? Can you give me a brief time line of your pain over the last 12 months?
Self and independence	Can you tell me about what things make you, you? What interests do you have, has this changed over the last year? What activities have you enjoyed over the last year? How would you describe yourself, has this changed over the last year? How has your pain affect how you feel about yourself over the last year? Can you tell me about the role of pain in terms of you from doing things that are important to you? Has this changed over the last year? Can you tell me about any ways your pain impacted your ability to make independent choices and decisions (eg, making a decision on your own about doing exercises or eating healthy or taking medication) over the last year? Can you tell me about how much freedom you have had over the last year to do the things you would like to do? Can you give me any examples of what this freedom is like? Can you tell me about times when you don't have as much freedom as you would like?
Future	What do you think your future looks like? Have your thoughts about the future changed over the last year? What things would you like to do over the next 5 years? Can you tell me about any ways you feel your pain may affect these plans?
Family/Home	Can you describe your family life to me? How has this changed over the last year? Who lives at home with you? How often do you see your parents/siblings (if not living with them) What does your relationship with your parents look like, and how has this changed over the last year? What does your relationship with your siblings look like, and how has this changed over the last year? What kind of activities do you do together with your parents/siblings? What do you tend to talk about with your parents/siblings? Do you feel like these relationships/activities/conversations have changed since the start of your pain? What things do you do at home, and how has this changed over the last year? Can you tell me about any chores you do? Thinking about other people your age, in what ways do you think your home life is similar/different? How do your family/housemates/partner react to your pain? How has this changed since we last spoke? Do you find this helpful/unhelpful, why/how? Can you tell me about how these things we have discussed about you at home have changed over the last year?
Friends and relationships	Now I would like to talk about friendships and other relationships. Can you tell me about any changes to your friendships over the last year? Can you tell me for you, what makes a good friend? How do you feel about your friendships, has this changed from how you felt a year ago? What do you like doing with your friends, and can you tell me about how this has changed over the last year? Can you tell me about any ways in which pain has influenced your friendships over the last 12 months? How do your friends react to your pain? How did you become friends with them? Can you tell me about any changes over the last year to how you spend time with your friends? What do you like doing with your friends? How do you communicate with your friends? (eg, social media) What does your relationship with friends look like when you are experiencing pain? How do your friends react when this happens? Can you tell me about any changes over the last year in how do you feel about your romantic relationships? Can you tell me about any ways in which your pain affects your relationships? How does your partner react to your pain? Can you tell me about any ways in which you feel your relationship is different from couples where chronic pain is not an issue?
School/Work	Can you tell me about school/education/employment over the last year? What changes have occurred since we last spoke? What kind of school/education/employment are you in? How do you feel you are doing at school/education/work? How do your teachers/employer react to your pain? How do your peers/colleagues react to your pain?
Close	Is there anything else you would like to tell me about your pain and your life? Are there any changes over the last year related to your pain and your life, that we haven't covered yet? Thank you for taking part in this study, your answers are very useful in helping us to better understand how pain impacts the lives of young people. That is everything for the study completed, thank you

## APPENDIX C

### ONLINE DIARY

#### Keeping on track: exploring socio‐developmental challenges faced by young people with chronic pain and their families

This online diary is where you can tell us about your experiences each month. We are keen to hear your thoughts as you are the experts when it comes to your pain and your lives. However, please remember you do not have to answer anything you do not want to, and you can decide to stop taking part in the study at any time. Please provide as much detail as possible, don't worry about spelling or how you write. Remember, that there are no right or wrong answers. While we want to hear about your life, we would appreciate it if you could try to avoid giving personal details of others where possible. This is because we don't have consent from those people to have their personal details. For example, if you are talking about your friends, you could say ‘my friend’ rather than their names.

What you write is confidential, which means we will not share it with anyone else (including your parents or your healthcare team). The only time we may have to share something is if you tell us something that makes us worried for your safety or the safety of someone else. If this happens, we will talk to you about who we need to talk to and why.

The questions are grouped into similar areas. You can choose to answer each question individually, or write an answer that answers all the questions in that group.

##### Family and home life

In this section, we are asking about your experience of family and home life over the past month.

- Can you describe your relationships with your close family over the past month?
- Can you tell us about how you have felt about your relationships with your family over the past month?
- What activities did you do with your family over the past month?
- How did you typically communicate with your family over the past month?
- What do you tend to talk about with your family in the past month?
- Can you tell us about any effects that your pain has had on these relationships over the past month?
- Can you tell us about any worries you had around your relationships with your family over the past month?

##### Friendships and social life

In this section, we are asking about your experience of friends and social life over the past month.

- What contact have you had with your friends over the past month? (eg, what activities, spending time together, phone calls, social media, seeing at school/university/work)
- Can you tell us about times over the last month when your pain got in the way of friendships, and in what ways?
- How have you felt about your friendships over the last month?

##### Education and work

In this section, we are asking about your experience of education and work life over the past month.

- Can you tell us about how things have been at school/university/work over the last month?
- How did you feel about yourself at school/university/work over the past month? This could include how much you enjoy your work, how much you feel you achieve at work, how much you feel part of a team, etc.
- Can you tell us about the activities you took part in at school/university/work over the past month?
- Can you tell us about any activities that you weren't able to take part in over the past month because of your pain?
- How were your relationships with your classmates/colleagues over the past month?
- Can you tell us about any worries you had about school/university/work over the past month?

##### Being you

In this section, we are asking about your experiences around your identity and sense of self over the past month.

- How do you feel that your pain has affected how you felt about yourself over the past month?
- What things did you enjoy doing over the past month? Can you tell us about any ways your pain affect things?
- How much control did you feel you had about what you do, and in making decisions about your life over the past month?
- Can you tell us about any ways your pain affected the control you had and decisions you made over the past month?
- What do you think about how your pain may affect your future day‐to‐day life?
- What do you think about how your pain may affect your future overall life plans?

##### Other areas

Is there anything else you would like to tell us around how you feel about yourself, your place in the world, your relationship with others, or how you feel you are developing, and how your pain affects this?

Thank you for completing this month's diary and sharing with us your experiences and insights. If you have any questions, please email kotstudy@bath.ac.uk. We will send you the next diary at the start of next month, but if there is something that you would like to tell us about between now and then, you can send it to us over email, on Facebook, on Twitter, or on Instagram.

If you have any questions about the study, you can contact the researcher on the details below. If this study has raised issues that you would like some support with, here are details for some organizations which may be able to help.

**Table:**

Researcher contact details	Abbie Jones (PhD Student at the University of Bath) A.jones@bath.ac.uk 01225 386982
Pain concern	www.painconcern.org.uk 0300 123 0789 (please see website for helpline opening hours)
Away with pain	www.awaywithpain.co.uk There is a specific section for young people with ongoing pain on the website
Samaritans	www.samaritans.org jo@samaritans.org Call: 116 123

## APPENDIX D

### EXAMPLE TEMPORAL MATRIX FOR THE FIRST SUBTHEME

**Table:**

Theme—The shifting sands of peer relationships' Participant—Ellie
**Baseline interview** “it [pain] has resulted in me being becoming quite distant from my friends”
**Diaries** Month 1 “I feel like my pain has put an awkward social barrier between me and my friends which has resulted in them not wanting anything to do with me anymore.” Month 5 “I went to a big party on new years eve with a lot of people in my year which I was very nervous about but I had a brilliant time. Now that the majority of my friend's are 18, they go out for drinks a lot so I've been sneaking into pubs with them and being ‘designated driver’ for them after! I'd say that pain hasn't got in the way of friendships this month.”
**Follow‐up interview** “My immediate friends were good and then it was like a couple of friends at school that were really great. Um, I did have a couple of groups that were quite difficult.”

**Table:**

Theme—The shifting sands of peer relationships' Participant—Laura
**Baseline interview** “I think that, before my pain I was like, more out there a bit, and I didn't really with where I went and what I did. Like I liked to go out with my friends and talk to everybody, whereas now I'm quite like a bit closed off and just stay in my room.”
**Diaries** Month 1 “I have been unable to go places with my friends for very long or anywhere outside of my house for very long really. I've been feeling really low over this month so I've distanced myself from my friends” Month 7 “Toward the end of my time out with the group I became tired and in a lot of pain so it was difficult to walk, which my friends took notice of and looked out for me when they did.”
**Follow‐up interviews** “My best friend has been really really good.”

**Table:**

Theme—The shifting sands of peer relationships' Participant—Joanna
**Baseline interview** “my friendship group changed, mainly because I wasn't in, either due to the IBS, or because of the pain. Which meant I had to find other people that would still, almost include me and wouldn't exclude me if I wasn't there.”
**Diaries** Month 4 “I had quite a bit of time off and sometimes got isolated from the conversations when I was in. I feel that my friendships have been good, but could be better if I was in school more.”
**Follow‐up interview** Joanna “Since last year, my friendship group has changed completely. People I used to be friends with, I'm still friends with but I don't hang around with. And I now have people who are a hell of a lot more supportive than they ever were.”
